# Loop mediated isothermal amplification: An innovative gene amplification technique for animal diseases

**DOI:** 10.14202/vetworld.2016.465-469

**Published:** 2016-05-11

**Authors:** Pravas Ranjan Sahoo, Kamadev Sethy, Swagat Mohapatra, Debasis Panda

**Affiliations:** 1 Department of Veterinary Biochemistry, Odisha University of Agriculture & Technology, Bhubaneswar, Odisha, India; 2 Department of Animal Nutrition, Odisha University of Agriculture & Technology, Bhubaneswar, Odisha, India; 3 Department of Veterinary Physiology, Odisha University of Agriculture & Technology, Bhubaneswar, Odisha, India

**Keywords:** designed primers, loop-mediated isothermal amplification assay, transboundary animal diseases

## Abstract

India being a developing country mainly depends on livestock sector for its economy. However, nowadays, there is emergence and reemergence of more transboundary animal diseases. The existing diagnostic techniques are not so quick and with less specificity. To reduce the economy loss, there should be a development of rapid, reliable, robust diagnostic technique, which can work with high degree of sensitivity and specificity. Loop mediated isothermal amplification assay is a rapid gene amplification technique that amplifies nucleic acid under an isothermal condition with a set of designed primers spanning eight distinct sequences of the target. This assay can be used as an emerging powerful, innovative gene amplification diagnostic tool against various pathogens of livestock diseases. This review is to highlight the basic concept and methodology of this assay in livestock disease.

## Introduction

India is a developing country, which economy is mainly contributed by the livestock sector. As there is more emerging of transboundary diseases, high molecular level research is being going on in India for devastating the economy loss [1]. For this purpose, nucleic acid amplification is one of the most valuable tools not only for the diagnosis of infectious diseases but also used in advanced gene level research. Traditional polymerase chain reaction (PCR) of its apparent high simplicity and reliability is mostly widely used as a standard approach in all biotechnological and medical diagnostic laboratories but due to its requirement for a high precision thermal cycler, it prevents this powerful method from being widely used such as in private clinics as a routine diagnostic tool. Other different modified amplification methods such as nucleic acid sequence based amplification, self-sustained sequence replication and strand displacement amplification (SDA) work efficiently with elimination some heat denaturation step without need of thermal cycler. First, two techniques have some compromise in sensitivity, which has to overcome and SDA overcomes this problem dueto the use of four primers and isothermal conditions for amplification but still has some disadvantage such as increased backgrounds due to digestion of irrelevant DNA contained in the sample and the necessity of costly modified nucleotides as substrate [2]. So, to overcome these above problems, another single-tube amplification technique should be developed that can amplify a few copies of DNA to 10^9^ in less than an hour under isothermal conditions with greater specificity. The loop-mediated isothermal amplification (LAMP) assay may fulfill all the above parameters for which it can be used as low-cost alternative for detection of different transboundary animal disease. This technique has the potential to revolutionize molecular biology because it allows DNA amplification under isothermal conditions and is highly compatible with point of care analysis [3]. So, this review may help the understanding of the basic principle, methodology and advances of LAMP assay in the livestock sector.

### Principle

LAMP is an isothermal nucleic acid amplification technique, in which amplification is carried out at a constant temperature without the need of thermal cycler. It is a powerful and novel nucleic acid amplification method, which detects the DNA at a very low level compared to other methods. This method amplifies very few copies of target DNA with high specificity, efficiency, and rapidity under isothermal conditions using a set of four specially designed primers and a DNA polymerase with strand displacement activity [4]. It has two steps of LAMP amplification comprising non cyclic and cyclic steps. In this process, two or three sets of primers are used specific to the target sequence which is amplified at a constant temperature of 60-65°C by a polymerase having high strand displacement activity. For identification of 6 distinct regions on the target gene, usually, 4 different primers are used which increases the specificity. Further use of “loop primers” cause increase rate the acceleration rate of the reaction for which the amplification is obtained within 30 min. The amount of DNA produced in this assay is considerably higher than traditional PCR based amplification due to the specific nature of these primers. Amplification and detection of gene are completed in a single step by incubating the gene sample, DNA polymerase, and substrates at constant temperature. In case of reverse transcription loop LAMP (RT-LAMP) assay, the process is initiated by reverse transcriptase from the backward internal primer (BIP), by binding to a target sequence on the 3′ end of the RNA template and synthesizing a copy DNA strand. Simultaneously, a new cDNA strand is created with the help of DNA polymerase by binding of the B3 primer to this side of the template strand while displacing the previously made copy. The single stranded copy now loops at the 3′ end as it binds to itself. The forward internal primer (FIP) binds to the 5’ end of this single strand and accompanied by DNA polymerase and synthesizes a complementary strand. The F3 primer, with DNA polymerase, binds to this end and generates a new double-stranded DNA molecule while displacing the previously made single strand. This new single strand that has been released, will act as the starting point for the LAMP cycling amplification. The DNA has a dumbbell like structure as the ends fold in and self anneal. This structure becomes a stem loop when the FIP or BIP primer once again initiates DNA synthesis at one of the target sequence locations. This cycle can be started from either the forward or backward side of the strand using the appropriate primer. Once this cycle has begun, the strand undergoes self-primed DNA synthesis during the elongation stage of the amplification process. This amplification takes place in only an hour, under isothermal conditions between 60°C and 65°C.

## Methodology

The whole procedure of this assay is very simple and rapid by incubating mixture of gene sample and six proper designed specific primers in a single tube with reverse transcriptase and Bst DNA polymerase at 63°C. The LAMP amplification includes a set of six primers comprising two outers, two internal and two loop primers that recognize eight distinct regions on the target sequence [5]. The primers are designed as two outer primers, such as forward outer primer (F3) and backward outer primer (B3), having strand displacement activity during the non-cyclic step only and also two internal primers, such as FIP and BIP, having both sense and antisense sequence which helps in loop formation. Further, two loop primers, i.e., forward loop primer and backward loop primer are designed to amplify the additional sites that are not accessed by internal primers. The amplified product is detected by agarose gel electrophoresis as well as by real time monitoring, which is based on photometry for turbidity. The turbidity is due to magnesium pyrophosphate precipitate which is being increasing in quantity as a byproduct of amplification. So, one can detect the amplification product by the naked eye whether it is of larger or smaller reaction volumes. The detection procedure can be done in real time either by measuring the turbidity or by fluorescence using intercalating dyes such as SYTO 9 [6]. A visible color change can be seen with naked eyes using dyes such as SYBR green. The dye molecules intercalate or directly label the DNA, which can be correlated with the number of copies making the LAMP be quantitative. Manganese loaded calcein can be used for detection of DNA amplification in the tube during *in vitro* DNA synthesis [2]. A small amount of low molecular weight polyethyleneimine is added to the LAMP reaction solution to detect LAMP products in a sequence-specific manner [7].

### Sensitivity and Specificity of LAMP

LAMP assay is found to be 10-100 fold more sensitive than PCR with a detection limit of 0.01-10 pfu of virus [8].

### LAMP in Diagnosis of Animal Diseases

Due to its simplicity, ruggedness, and low cost, LAMP has the potential to be used as a simple screening assay in the field or at the point of care by veterinarian [9]. This assay without the use of expensive thermocyclers is being used for diagnosis infectious disease in developing countries [10]. In the medical field, LAMP is widely being studied for detecting infectious diseases such as tuberculosis [11], malaria [12] and sleeping sickness [13]. LAMP has been successfully applied for rapid and real time detection of both DNA and RNA viruses. A one step single tube real time accelerated RT-LAMP assay has been developed for rapid detection of each of several recently emerged human viral pathogens i.e., Dengue, Japanese encephalitis, Chikungunya, West Nile, SARS, highly pathogenic avian influenza H5N1, and Norwalk viruses [14-18]. Amplification of DNA viruses, such as human papillomavirus type 6, 11, 16, and 18, was done by LAMP due to its high sensitivity, specificity, rapidity, and simplicity [19]. Although this assay has been started in the veterinary field, it needs to be concerned. LAMP assays that have been developed for the detection of 18 viruses deemed notifiable of ruminants, swine, and poultry by the World Organization for Animal Health [20]. Among the animal viruses, a one-step, RT-LAMP assay has been developed for detection of foot and mouth disease virus in less than 1 h in a single tube without thermal cycling [21]. This assay is done by amplifying a fragment of the 3D RNA polymerase gene at 65°C in the presence of primer mixture, reverse transcriptase, and Bst polymerase. Similarly, a single step RT-LAMP was developed against G-protein sequence for detection of viral hemorrhagic septicemia virus [22]. A set of primers designed against on canine parvovirus in fecal sample, a LAMP has been reported by Cho *et al* [23]. A new of LAMP assay coupled with lateral flow dipstick for the detection of classical swine fever virus has been developed by Chowdry *et al* [24]. This assay was developed against *Coxiella burnetii* targeting the *com1* gene as an actual alternative to conventional PCR [25]. LAMP test targeting the *p40* gene of *Mycoplasma agalactiae*, for the diagnosis of classical contagious agalactia was developed by Rekha *et al* [26]. This test for detection of *Mycoplasma synoviae* strains in poultry using specifically designed primers targeting *hemagglutin A* (vlh) gene was developed by Kursa *et al* [27]. This assay for specific and rapid detection of *Brucella abortus* was developed from clinical samples of cattle [28]. This assay was found to be useful in the detection of Marek’s disease in feathers and internal organs of infected chickens [29]. One step RT-LAMP method for rapid detection of the hemagglutinin and neuraminidase genes of H7N9 virus was developed Zhang *et al* [30]. A LAMP assay with 6 primers targeting a highly conserved region of the GRA1 gene was developed to diagnose *Toxoplasma gondii* [31]. It has been emerged as a novel nucleic acid amplification method for diagnosis of visceral leishmaniasis [32]. The assay with the best performance was targeted to the egl gene, which shows high analytical specificity for diverse strains of the beta proteo bacterium *Ralstonia solanacearum* [33]. This assay is currently used as standalone diagnostic test for *Clostridium difficile* infection [34]. In addition to above developments, LAMP assay can be extensively applied in the field of molecular diagnosis of cancer, identification of genetically modified organisms, detection of food adulteration, eutrophication, food allergens, pesticides, identification of medicinal plants, drug resistance, and DNA methylation studies [35].

### Advantages of LAMP

Due to its ability to amplify nucleic acid under isothermal conditions in the range of 65°C, it only needs simple and low cost effective equipment. LAMP stands out to be a good and effective diagnostic test for empowering in developing countries as it does not require sophisticated equipment and skilled personnel and proves to be cost effective [36]. Its specificity is extremely high because it can amplify a specific gene by discriminating a single nucleotide difference [37]. Its amplification efficiency of LAMP is very high because there is no time loss of thermal change. The enzyme inhibition reaction at the later stage of amplification is less likely to occur because this assay is being undergoing at optimum temperature of the enzyme. The nucleic acid amplified by the LAMP method is detected through the naked eye by observing the turbidity derived from the precipitate. It can be visually detected through fluorescence by utilizing calcein in loop amp fluorescent detection reagent [2]. The fluorescence is generated by binding of pyrophosphate ions, i.e., the by-product of the amplification, to the manganese ions from calcein. The fluorescence is further intensified as calcein combines with magnesium ions. It involves both amplification and detection of the gene in a single step, by incubating the mixture of gene sample, primers, DNA polymerase with strand displacement activity and substrates at a constant temperature. Due to its high amplification efficiency, DNA can be amplified 10^9^-10^10^ times in 15-60 min. There is no need denaturation step i.e., conversion of double-stranded DNA into a single stranded form, so it requires less time. LAMP assay has the great advantage of monitoring amplification by SYBR Green I dye mediated naked eye visualization and by real-time monitoring using an inexpensive turbid meter according to the situation [38].

### Disadvantages of LAMP

Although LAMP has above advantages, it has some following disadvantages. Kikuchi *et al*. stated that it is more sensitive and specific than PCR as [39], but it seems to be less sensitive than PCR to inhibitor in case of complex samples such as blood, likely due to the use of a *Bst* DNA polymerase rather than *Taq* polymerase as in PCR [40]. It is less versatile than PCR. LAMP is useful primarily as a diagnostic or detection technique but not useful for cloning purposes. Proper designing of primer is a major constraint in this assay [41]. Multiplexing approaches for LAMP are less developed than for PCR. The larger number of primers per target in LAMP increases the primer-primer interactions. The product of LAMP is a series of concatemers of the target region, giving rise to a characteristic “ladder” or banding pattern on a gel, rather than a single band as with PCR.

## Conclusion

LAMP is an innovative, new generation, gene amplification technique that can amplify the target sequence with a high degree of sensitivity and specificity under isothermal condition. The invention of this method a decade ago has given new impetus toward the development of point of care diagnostic tests based on amplification of pathogen DNA, a technology that has been the precinct of well developed laboratories [42]. Due to its easy detection procedure, i.e., on real-time nucleic acid amplification, this assay can be used as pen side point of care diagnostic tool for infectious animal disease. Thus, it will provide a great platform for quick and accurate identification of different pathogens in medical as well as veterinary field.

## Authors’ Contributions

Each and every author has contributed the relevant literature in preparation of this work of review. PRS carried out his investigations and experimentations on the mentioned topic. KS searched various related topics for better reference purpose. SM corrected the grammatical errors exists in the manuscript and DP designed the proper format of the manusript. All authors read and approved the final manuscript.
